# High-Contrast Visualization Chemiluminescence Based on AIE-Active and Base-Sensitive Emitters

**DOI:** 10.3390/molecules28093976

**Published:** 2023-05-08

**Authors:** Xiao-Wen Zhang, Xu-Lin Chen, Can-Zhong Lu

**Affiliations:** 1 State Key Laboratory of Structural Chemistry, Fujian Institute of Research on the Structure of Matter, Chinese Academy of Sciences, Fuzhou 350002, China; 2 Fujian Science & Technology Innovation Laboratory for Optoelectronic Information of China, Fuzhou 350108, China; 3 Xiamen Key Laboratory of Rare Earth Photoelectric Functional Materials, Xiamen Institute of Rare Earth Materials, Haixi Institutes, Chinese Academy of Sciences, Xiamen 361021, China; 4 School of Physical Science and Technology, Shanghai Tech University, Shanghai 201210, China

**Keywords:** chemiluminescence, aggregation-induced emission, base-sensitive, high-contrast visualization, encryption and decryption

## Abstract

Peroxyoxalate chemiluminescence (PO-CL) is one of the most popular cold light sources, yet the drawback of aggregation-caused quenching limits their use. Here, we report a new kind of efficient bifunctional emitter derived from salicylic acid, which not only exhibits typical aggregation-induced emission (AIE) character but also has the ability to catalyze the CL process under basic conditions based on base sensitivity. By taking advantage of these unique features, we successfully confine the CL process on the surface of solid bases and provide a high-contrast visualization of CL emission. This method allows most of the common basic salts like sodium carbonate to be invisible encryption information ink and PO-CL solution to be a decryption tool to visualize the hidden information. The current study opens up an appealing way for the development of multifunction CL emitters for information encryption and decryption applications.

## 1. Introduction

Chemiluminescence (CL) is a type of light radiation that occurs when substances undergo chemical reactions without light, heat, sound, electricity, or magnetism to trigger. Due to the comparatively mild reaction mechanism, CL can provide a relatively long-sustained environmental-friendly cold light source, which has been widely used as an intuitive visualization method in many fields such as flow testing [1,2,3,4], biological imaging [5,6,7,8], environmental detection [9,10,11], etc. The peroxyoxalate chemiluminescence (PO-CL) system is one of the most popular CL branches that share advantages of high brightness, long duration, high compatibility, and low cost. A PO-CL system is composed of four compounds: oxalate, fluorescer, oxidant, and catalyst [12]. Through component selection, the PO-CL system can change luminescence intensity, duration, and color, making it suitable for high-precision applications such as liquid chromatography [13,14,15].

In the PO-CL system, the catalyst plays a role as a booster that accelerates the decomposition reaction of peroxyoxalate, resulting in a shorter but brighter CL process [16,17,18]. The catalyst needs to be basic and soluble in organic solvents to increase the speed with which the ester bond breaks down. Salicylate [17] is one of the common catalysts in the PO-CL systems. Due to its phenolic hydroxyl group and weak basicity, salicylate has a low first electron oxidation potential, making it easier to catalyze the conversion of intermediate peroxides into free radicals. Therefore, it can be inferred that fluorescers with salicylate structure can act as an emitter and a high-activity catalyst simultaneously in PO-CL systems.

Aggregation-induced emission (AIE), a photophysical phenomenon associated with chromophore aggregation, is another a hot research topic in the material science field [19]. AIE-active luminogens can be realized by restriction of intramolecular rotation (RIR) and intramolecular vibration (RIV) [19,20]. Tremendous research interests have been attracted to develop enormous varieties of AIEgens for diversified applications, such as fluorescent chemosensors [21,22], imaging contrast agents [23,24,25], electrochemiluminescence luminophores [26], and organic light-emitting diodes materials [27,28,29,30,31]. Tang [8] introduced the AIE group in the luminol–chemiluminescence system and achieved a high-contrast tissue image. By linking the tetraphenylethene (TPE) group with cationic surfactants, Lu [32] successfully controlled the aggregation-caused quenching problem of luminol. However, till now, AIE materials have rarely been used in PO-CL systems, although AIE activities of CL emitters have the potential to realize high-contrast images. One of the reasons is that the small molecule AIE fluorophores are hard to meet the demand of high-speed CL resonance energy transfer. The other is that AIE fluorophores behave with poor luminous efficiency in low aggregate states, while the PO-CL process mainly occurs in solution.

**Figure 1:**
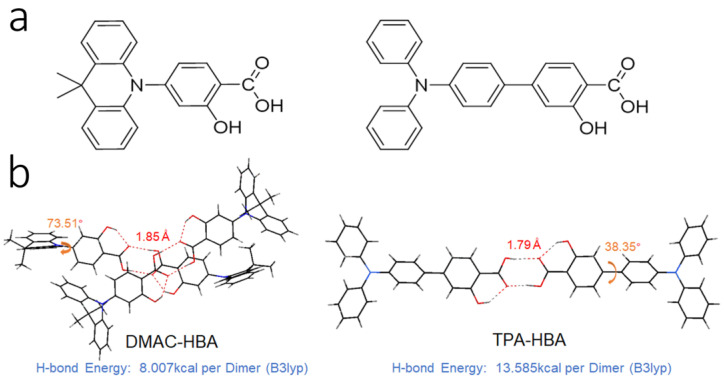
(**a**) Chemical structures and (**b**) crystal structures of DMAC-HBA and TPA-HBA. Hydrogen bonds are indicated in red and torsion angles are indicated in orange.

Herein, we report two fluorophores, DMAC-HBA and TPA-HBA (Figure 1a), which are comprised of 9,9-dimethyl-9,10-dihydroacridine (DMAC) or triphenylamine (TPA) as electron-donor and the salicylic acid group as electron-acceptor. The salicylic acid unit endows these materials with a unique sensibility to Lewis-base and good solubility in various organic solvents. Moreover, these emitters show unique water-caused-quenching (WCQ) and AIE behaviors in both water/THF and PO-CL systems. This new emitter can not only be used as an emitter in the PO-CL system but also play a catalytic role, such as sodium salicylate, in the oxidation process of peroxyoxalate under alkaline conditions. By utilizing the chemisorption of salicylic acid emitters on the surface of a basic solid, we successfully introduced the AIE phenomenon into the PO-CL system and created high-contrast visualization of CL patterns.

## 2. Results and Discussion

### 2.1. Synthesis and Characterization

4-(9,9-dimethylacridin-10(9H)-yl)-2-hydroxybenzoic acid (DMAC-HBA) was synthesized from 4-bromo-2-hydroxybenzoic acid and dimethylac-ridin (DMAC) via Buchwald–Hartwig coupling reactions. 4′-(diphenylamino)-3-hydroxy-[1,1′-biphenyl]-4-carboxylic acid (TPA-HBA) was synthesized via Suzuki coupling from 4-bromo-2-hydroxybenzoic acid and 4-(diphenylamino)phenylboronic acid. The final products were purified through column chromatography on silica gel and then identified by ^1^H NMR and ^13^C NMR spectra. The synthetic routes are outlined in Figure S1, and detailed synthesis procedures are described in Section 3 in detail.

### 2.2. Photophysical Properties

**Figure 2:**
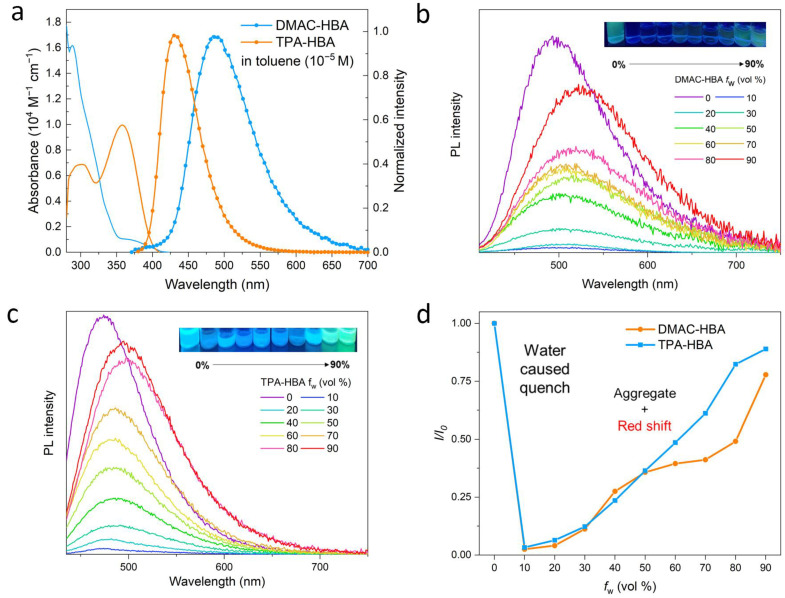
(**a**) UV/Vis absorption and photoluminescence (PL) spectra of DMAC-HBA and TPA-HBA in toluene (c = 10^−5^ M) at room temperature; PL spectra of DMAC-HBA (**b**) and TPA-HBA (**c**) in THF/H_2_O mixture with different water fraction (f_W_); (**d**) Variation in relative PL intensity (*I*/*I*_0_) of DMAC-HBA and TPA-HBA with different f_W_, where *I*_0_ is the initial PL intensity measured in THF solution. The PL measurements were excited at 410 and 420 nm for DMAC-HBA and TPA-HBA, respectively.

**Figure 3:**
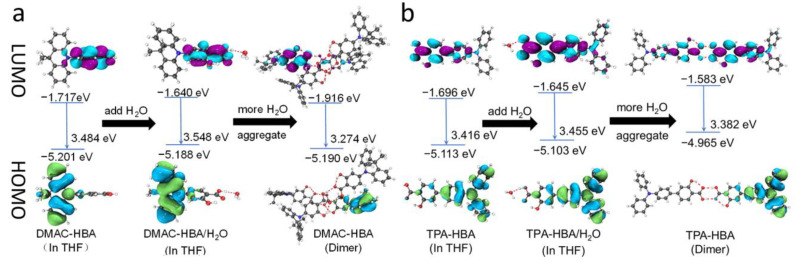
Frontier molecular orbitals of (**a**) DMAC-HBA and (**b**) TPA-HBA in different states.

The UV-vis absorption and photoluminescence (PL) spectra of DMAC-HBA and TPA-HBA in dilute toluene solutions are shown in Figure 2a. Although these compounds show similar donor (D)-acceptor (A) structures, these absorption and emission spectra at room temperature are obviously different, implying the remarkable difference in the electronic transition nature of the singlet excited states. DMAC-HBA displays intense absorption bands below 350 nm and a weaker absorption shoulder (350–420 nm, ε ≈ 10^3^ M^−1^ cm^−1^), which can be assigned to spin-allowed π–π* transition and spin-forbidden CT transition, respectively. In comparison, the low-energy absorption band of TPA-HBA corresponding to the HOMO–LUMO transition is much more intense (ε ≈ 10^4^ M^−1^ cm^−1^) than the CT absorption band of DMAC-HBA, indicating that HOMO–LUMO transition of TPA-HBA probably possesses hybrid local and CT (HLCT) nature owing to the large frontier molecular orbital (FMO) overlaps (vide infra). The toluene solutions of DMAC-HBA and TPA-HBA exhibit cyan (λ_max_ = 506 nm) and deep-blue (λ_max_ = 435 nm) emission with structureless spectrum profiles, respectively. In contrast to DMAC-HBA, TPA-HBA shows a much narrower emission spectrum (full width at half maximum: 51 nm vs. 79 nm) and a much smaller Stokes shift (73 nm vs. 202 nm), which could be attributed to the significant local excitation (LE) contributions in the emissive singlet state of TPA-HBA. According to theoretical calculations (Figure 3), compared with DMAC-HBA, TPA-HBA in dilute solution shows a much smaller D-A torsion angle (33.06° vs. 82.02°), resulting in a larger HOMO–LUMO overlap. This is also the main reason why TPA-HBA shows a larger transition dipole moment and more efficient PL. To further figure out the PL mechanism, the transient PL decay curves of these compounds in toluene before and after Ar bubbling were recorded. As shown in Figure S8, the as-prepared solution of each compound showed only a prompt decay component. After removing the dissolved oxygen by Ar bubbling, the decay curve of TPA-HBA showed nearly no change, while an additional new delayed decay component appeared in the decay curves of DMAC-HBA, which should be thermally activated delayed fluorescence (TADF) emission.

These two compounds are very soluble in tetrahydrofuran (THF) but completely insoluble in water. Dilute THF solutions of DMAC-HBA and TPA-HBA are efficiently emissive with spectrum maxima at 498 nm and 475 nm, respectively, while they undergo interesting changes in emission properties when water is gradually added into these solutions. As shown in Figure 2b,c, take TPA-HBA for example, the strong emission of THF solution can be dramatically quenched with a small amount of water (water fraction f_w_ = 10 vol%), accompanied by blue-shifting the emission maximum to 470 nm. While further increasing f_w_, the emission wavelength gradually redshifted with emission intensity gradually enhanced. Similar phenomena were observed for DMAC-HBA, although the PL intensity of each DMAC-HBA sample was weaker than its TPA-HBA counterpart. To find out the reasons behind these observations, we investigated the single-crystal structures and performed theoretical calculations. As shown in Figure 1b, the C=O…H-O distances between adjacent carbonyl and hydroxyl groups on the salicylic acid units of DMAC-HBA and TPA-HBA are as short as 1.85 Å and 1.79 Å, respectively, indicating strong intramolecular and intermolecular hydrogen bonds. In dilute THF solutions, the intramolecular hydrogen bonds in each molecule, which makes the salicylic acid unit form a rigid electron-acceptor plane, are expected to minimize the non-radiative relaxation and facilitate radiative D–A transitions and thus ensure efficient PL. At the same time, a certain amount of water molecules adding to dilute THF solutions would compete to form intermolecular hydrogen bonds with the salicylic acid units of DMAC-HBA and TPA-HBA (Figure 3). On the one hand, the non-radiative decay would be aggravated owing to the bond free rotation and vibration. On the other hand, according to the density functional theory (DFT) calculations (Figure 3), the newly formed intermolecular hydrogen bonds between the carbonyl and H_2_O weaken the acceptor strength of the salicylic acid unit and, in turn, increase the HOMO–LUMO energy gap. These points could rationalize the observed emission quench and blue-shift when adding a small amount of water to the THF solution of these compounds. When the water fractions further increase, the AIE mechanism takes effect. The high water fractions in the mixtures cause the formation of nanoaggregates, where the physical dimers (Figure 1) are formed via intermolecular hydrogen bonding of the salicylic acid groups. These intermolecular hydrogen bonds restrict molecular rotatory and vibratory motions, enabling RIR and RIV to take effect to enhance light emission. The DFT and time-dependent DFT (TD-DFT) calculations (Figure 3 and Figure S6) reveal that the physical dimers of these molecules possess lower-energy S_1_ states with significant intermolecular CT characteristics and larger oscillator strengths compared with those of the monomers.

### 2.3. Chemiluminescence and Base-Sensitivity

**Figure 4:**
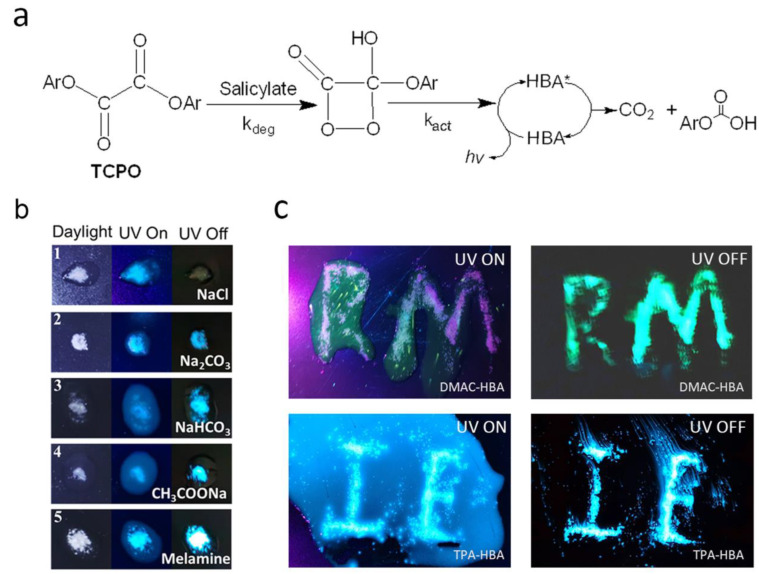
(**a**) General CL process of the PO-CL system based on bis(2,4,6-trichlorophenyl) oxalate (TCPO) (Ar: 2,4,6-trichlorophenyl), HBA is the emitter. * indicates excited state. (**b**) Emission photos of TPA-HBA under daylight, 365 nm-UV light (PL), and dark field (CL) when the TPA-HBA-based PO-CL solution is dropped onto different powders (1. sodium chloride; 2. sodium carbonate; 3. sodium bicarbonate; 4. sodium acetate; 5. melamine). (**c**) Emission photos of PO-CL solution based on DMAC-HBA (up) or TPA-HBA (down) on patterning sodium carbonate powders when UV on (left, PL) and UV off (right, CL).

As illustrated in Figure 4a, in the PO-CL systems, salicylates are usually used as catalysts to catalyze the oxalate ester TCPO (bis(2,4,6-trichlorophenyl) oxalate) to generate an energy-rich peroxide intermediate. In order to better utilize the excitons generated by TCPO, the activators (ACT) are considered to require high fluorescence quantum yield. Most of the neutral activators involved in the electron translation process can boost the k_act_ with no influence on the decomposition process, which means that the k_deg_ is only affected by the catalyst, such as salicylate. Several studies [1,23] have found that the hydro-abundant surface, like anatase or surfactant, can play a role as a catalyst to increase k_deg_ for sensing oxygen. Salicylate catalysts can play a key role in the PO-CL system to determine the reaction rate of this step and, in turn, affect the CL intensity and duration time. Since TPA-HBA and DMAC-HBA belong to salicylic acid derivatives, it can be inferred that they have the same ability to catalyze the CL process as salicylate under basic conditions, in addition to the emitter role. Moreover, considering their Lewis acidity and AIE character, these salicylic acid emitters are expected to enrich and aggregate on the solid base surface via chemical adsorption and then show enhanced emission intensity relative to those in solution.

Based on the above considerations, we explored the effect of salt and base solids on the PL and CL processes of these salicylic acid emitters by dropping TPA-HBA-based PO-CL solution onto salt and base powders, including sodium chloride, sodium carbonate, sodium acetate, sodium bicarbonate, and melamine, respectively. As shown in Figure 4b, under ultraviolet light, the PL intensities of TPA-HBA were obviously enhanced on all the solid surfaces compared with those in the solutions, which are in accord with the AIE behaviors observed in the THF/H_2_O system and can be attributed to the molecular aggregation on the surfaces of solid salt/base. After removing the UV source, all the base-treated samples showed bright CL on the solids, where the CL intensity had no obvious correlation with the basicity of base solids, while the NaCl-treated one showed no CL. Importantly, the CL on the base solids was even brighter than the corresponding PL, while solutions around the base solids exhibited no CL, resulting in high-contrast visualization of CL (Figure 4b,c). These observations suggest that the salicylic acid emitters are base-sensitive and that a base is indispensable for them to generate CL. We speculate that the salicylic acid molecules react with the base on the solid surface and yield salicylates or acid-base complexes, which act as a catalyst in the CL process. Notably, the melamine-treated sample showed the strongest PL and CL enhancement among the five base-treated samples, which may be attributed to its solubility and strong hydrogen bond adsorption capacity of the amino group to the carboxyl group. In conclusion, based on their unique molecular structures and base sensitivity, these salicylic acid molecules play a dual role, namely an emitter and a catalyst, in the CL process. Meanwhile, the AIE character ensures these salicylic acid emitters realize high-contrast visualization of CL.

**Figure 5:**
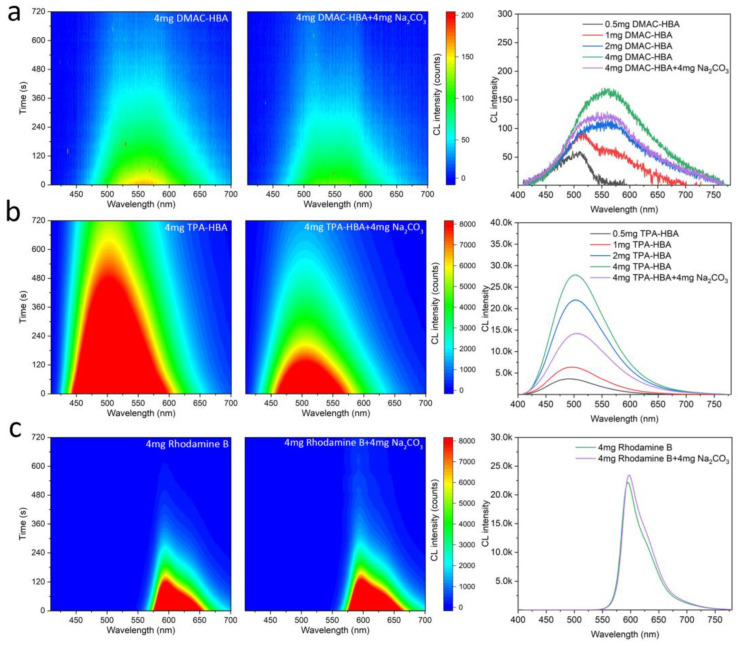
Contour maps of chemiluminescence spectra of the PO-CL solutions consisting of 4 mg emitter (left) and 4 mg emitter + 4 mg solid Na_2_CO_3_ (mid); chemiluminescence spectra of the PO-CL solutions of various emitter concentrations (right), where the CL emitters are DMAC-HBA (**a**), TPA-HBA (**b**), and Rhodamine B (**c**), respectively.

Although the above experiments proved that bright CL occurred on the solid surface, the influence of base solid on the CL process in solution is still unclear. To further explore the influence of base in the PO-CL system with DMAC-HBA or TPA-HBA as fluorescent emitters, we testified the CL process with sodium salicylate as a catalyst in the test and Rhodamine B as the control emitter. With the collection by a luminance meter, the time-dependent CL intensities of samples with different emitter concentrations were recorded. In order to prepare the material for testing, we divided it into A liquid and B liquid. Liquid A consists of TCPO, dibutyl phthalate (DPB), and emitter; Liquid B consists of tert-butanol, hydrogen peroxide, and sodium salicylate (see Supporting Information for details). In fact, without adding sodium salicylate as a catalyst, no CL can be observed in the PO-CL solution, indicating that DMAC-HBA and TPA-HBA cannot directly act as a catalyst for the CL process. With adding sodium salicylate as the catalyst, the CL intensities of the PO-CL solutions based on DMAC-HBA and TPA-HBA emitters are obviously enhanced with the increase of emitter concentration (Figure 5). The additional sodium carbonate did not change the wavelength of CL of both salicylic emitters but decreased the CL intensity and duration time in the PO-CL solution. In contrast, Rhodamine B did not show a decrease in CL intensity and duration time. These results suggest that sodium carbonate is not a direct catalyst for chemical reactions in the PO-CL system but rather acts as a Lewis base and provides a phase-separated interface to adsorb the salicylic acid emitters, forming catalytic acid-base complex on the solid surface. Due to the insolubility of sodium carbonate in DPB solution, the sodium carbonate deposited at the bottom of the DPB solution absorbs part of the HBA fluorescents, resulting in lowering the concentration of HBA fluorescents in the solution and reducing the CL intensity in the solution phase. In the condition without adding sodium salicylate, therefore, the system only emits bright CL on the surface of the solid base and no CL in the solution, resulting in high-contrast visualization CL as shown in Figure 4c.

### 2.4. Information Encryption and Decryption Application

**Figure 6:**
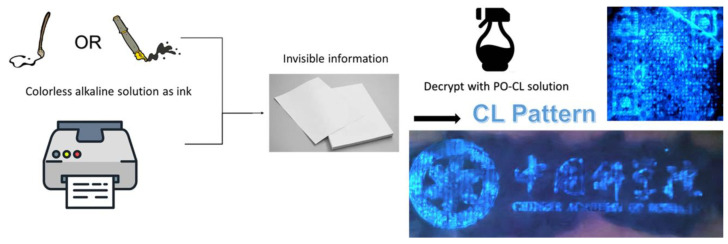
Schematic illustrations of the information encryption and decryption process using TPA-HBA-based PO-CL system. The chemiluminescence images show a QR code and the CAS badge using ink-jet printing.

Based on the above results, we expected that solid-state Lewis bases could be introduced as probes for these PO-CL systems. Benefiting from the AIE performance and base-conversion strategy, our fluorophores probably have great potential to realize confidential information encryption and decryption applications. At present, almost all encryption methods in the visible range are based on PL on/off, which means that emitters need to be printed on paper and can be easily observed. Obviously, it is easy to find the location of the encrypted information through naked-eye observation or simply ultraviolet light irradiation, which means that the confidentiality of the encrypted location itself is very poor. According to the enhancement effect of base salt such as carbonate or acetate on the CL, the encrypted information can be written in an invisible way by simply using a saturated base salt solution as the transparent ink. As shown in Figure 6, we wrote or printed the information that needed to be encrypted on paper with saturated sodium carbonate aqueous solution, and when the sodium carbonate dried naturally, it would naturally solidified on the surface of the paper. Then, we sprayed the prepared salicylic acid emitter-based-PO-CL solution on the paper, and the information can be displayed in the form of visible to the human eyes through chemiluminescence. This CL encryption method shows high-contrast visualization and has no color and no smell to be found.

## 3. Experiments

### 3.1. General Procedures

All reactions were performed under N_2_ atmosphere using standard Schlenk unless specified. Solvents were freshly distilled over appropriate drying reagents. Bis(2,4,6-trichlorophenyl) oxalate (TCPO, 98%), rhodamine B (C_28_H_31_ClN_2_O_3_, 98%), dibutyl phthalate (DBP, 98%), 1,3,5-triazine-2,4,6-triamine (melamine, 98%), all the sodium salts, and all the chemicals for synthesizing DMAC-HBA and TPA-HBA were commercially purchased from Shanghai Bide Co., Ltd. (Shanghai, China) and used without further purification. ^1^H NMR and ^13^C NMR spectra were recorded on a Bruker Avance III NMR spectrometer in deuterated chloroform (CDCl_3_). X-ray diffraction data of these compounds were collected on a D8 VENTRUE diffractometer from Bruker, Karlsruhe, German. UV-Vis absorption spectra were recorded under ambient conditions with an Cary 5000 UV-Vis spectrophotometer from Agilent, Santa Clara, CA, USA. Steady-state. PL spectra were measured using a Xenon lamp as an excitation light source on an Edinburgh FLS980 from Edinburgh Instruments, Livingston, UK. The transient PL decay curves were measured on the same spectrophotometer (FLS980) in time-correlated single-photon counting (TCSPC) mode with an NT242-1K OPO laser as an excitation light source from EKSPLA, Vilnius, Lithuania. Contour maps of CL spectra were collected on a A1-MP spectrophotometer from Nikon, Tokyo, Japan.

### 3.2. Synthesis of Materials

#### 3.2.1. Synthesis of 4-(9,9-Dimethylacridin-10(9H)-yl)-2-hydroxybenzoic Acid (DMAC-HBA)

3.0 mmol of 4-bromo-2-hydroxybenzoic acid (0.648 g, 3 mmol), 3.0 mmol of 9,9-dimethyl-9,10-dihydro- acridine (0.627 g, 3 mmol), cesium carbonate (3.91 g, 12.00 mmol), and tri-tertbutylphosphonium tetrafluoroborate (131 mg, 0.45 mmol) were dissolved in dry toluene (60 mL). After adding palladium (II) acetate (34 mg, 0.15 mmol), the reaction mixture was stirred at 120 °C for 12 h under a nitrogen atmosphere. After the reaction was completed, the crude product was added excessive hydrochloric acid and concentrated with brine and CH_2_Cl_2_. The collected organic phase was dried over anhydrous Na_2_SO_4_ and concentrated by rotary evaporation. The crude product was purified by column chromatography on silica gel to give a yellow-green powder (yield: 67.6%). ^1^H NMR (600 MHz, CDCl_3_) δ 10.56 (m, 1H), 8.09 (dd, *J* = 8.5, 4.1 Hz, 1H), 7.48 (dt, *J* = 7.8, 1.9 Hz, 2H), 7.51–7.44 (m, 2H), 7.14–6.98 (m, 5H), 6.94 (ddd, *J* = 8.5, 5.1, 2.0 Hz, 1H), 6.65 (dd, *J* = 13.6, 8.0 Hz, 2H), 1.65 (s, 6H). ^13^C NMR (151 MHz, CDCl_3_) δ 164.26, 140.20, 126.46, 125.20, 122.29, 122.19, 36.61, 36.57, 30.29, 30.22.

#### 3.2.2. Synthesis of 4′-(Diphenylamino)-3-hydroxy-[1,1′-biphenyl]-4-carboxylic Acid (TPA-HBA)

3.0 mmol of 4-bromo-2-hydroxybenzoic acid (0.648 g, 3 mmol), 3.0 mmol of (0.867 g, 3 mmol), Pd(PPh_3_)_4_ (57.8 mg, 0.05 mmol) and K_2_CO_3_ (0.818 g, 6 mmol) were dissolved in THF/H_2_O solution (15 mL/3 mL). After evacuating and purging with nitrogen gas three times, the mixture was refluxed at 70 °C for 12 h under a nitrogen atmosphere. After cooling to room temperature, the crude product was added excessive hydrochloric acid and concentrated with brine and CH_2_Cl_2_. The collected organic phase was dried over anhydrous Na_2_SO_4_ and concentrated by rotary evaporation. The crude product was purified by column chromatography on silica gel to give a lime-green powder (yield: 80.4%). ^1^H NMR (600 MHz, CDCl_3_) δ 10.47 (s, 1H), 7.93 (d, *J* = 8.3 Hz, 1H), 7.50 (d, *J* = 8.7 Hz, 2H), 7.28 (t, *J* = 7.9 Hz, 4H), 7.20 (d, *J* = 1.7 Hz, 1H), 7.18–7.10 (m, 7H), 7.06 (t, *J* = 7.3 Hz, 2H). ^13^C NMR (151 MHz, CDCl_3_) δ 174.00, 162.53, 149.19, 147.39, 132.47, 131.33, 129.49, 128.02, 125.01, 123.57, 123.00, 118.02, 115.00, 109.46.

### 3.3. Preparation of CL Systems

#### 3.3.1. Preparation of CL Systems

The salicylate-based CL systems were prepared as follows: the CL solution was formed by mixing Solution A and Solution B. Solution A consisted of 0.4 g of bis(2,4,6-trichlorophenyl) oxalate powder dissolved in 10 mL of dibutyl phthalate, and 1 mL of a fluorescent dye solution with different concentration gradients (DMAC-HBA, TPA-HBA, and rhodamine B at 0.5, 1, 2, and 4 mg mL^–1^). Solution B consisted of 0.01 g of sodium salicylate in 2 mL of 30% H_2_O_2_ solution and 8 mL of dibutyl phthalate. After evenly mixing Solution A and Solution B, bright CL could be observed with the naked eye. The CL photos were collected by a digital camera, and the CL spectra were recorded by a system comprising a spectrophotometer during continuous CL emission.

#### 3.3.2. Preparation of Encryption Inks/Decryption Solutions

Colorless alkaline aqueous solutions such as sodium carbonate solution or melamine solution can be used as colorless encryption inks (unseen after dryness) for pens and printers. These encryption inks are compatible with most commercial inks. The decrypted solutions are consistent with the PO-CL solutions, except that sodium salicylate is not added as a catalyst.

### 3.4. X-ray Crystallographic Analysis

X-ray diffraction data of DMAC-HBA and TPA-HBA were collected on a Bruker D8 VENTRUE diffractometer equipped with graphite-monochromated Mo Kα radiation (λ = 0.71073 Å). Structures were solved by direct methods and refined by full-matrix least-squares methods with the SHELXL-97 program package. Hydrogen atoms were added in idealized positions. All non-hydrogen atoms were refined anisotropically. Details of crystal and structure refinement are listed in Tables S1–S5 in the Supporting Information. CCDC 2180970 and 2180971 contain supplementary crystallographic data for DMAC-HBA and TPA-HBA.

### 3.5. Computational Methodology

Theoretical simulations were performed using the Gaussian 09 program packages. The distributions and energy levels of FMOs were obtained by DFT calculations at the B3LYP/def2-SVP level. The excited states of target molecules were calculated by TD-DFT procedure employing range-separated exchange density functionals at the PBE0/def2-SVP level. The FMO distributions were visualized using Gaussview and VMD.

The atomic coordinates of the solid-state were obtained by X-ray single crystal diffraction, and the single excited state and triple excited state of the compound were further optimized. The hydrogen bond energies were calculated by B3LYP-D3(BJ) based on the length of hydrogen bonds according to the single crystal structures. The molecular configurations in solution are the result of optimization simulation in pure THF environment. The distances between H_2_O and the investigated molecules in the solution state were simulated by B3LYP. The atomic coordinates of the dimer were obtained by X-ray single-crystal diffraction without further optimization.

## 4. Conclusions

In summary, we have designed and synthesized two salicylic acid-derived donor-acceptor fluorophores. These newly designed salicylic acid-based materials show typical AIE activity and can react with Lewis-base to form salicylate catalysts for CL processes. Based on such unique properties, these fluorophores play a dual role, namely an emitter and a catalyst, in the PO-CL systems and realize CL with high-contrast visualization. Eventually, by using the soda solution as the invisible ink and the salicylic acid emitter based-PO-CL solution as the developer, confidential information encryptions and decryptions have been successfully realized. We anticipate that this study will inspire the development of a new class of multifunction luminescent materials for CL applications.

## Data Availability

All the data generated by this research are included in the article.

## Supplementary Materials

The following supporting information can be downloaded at: https://www.mdpi.com/article/10.3390/molecules28093976/s1, Figure S1. ^1^H-NMR spectrum of DMAC-HBA (600 MHz, CDCl_3_); Figure S2. ^13^C-NMR spectrum of DMAC-HBA (600 MHz, CDCl_3_); Figure S3. ^1^H-NMR spectrum of TPA-HBA (600 MHz, CDCl_3_); Figure S4. ^13^C-NMR spectrum of TPA-HBA (600 MHz, CDCl_3_); Table S1. Crystal data and structure refinements for DMAC-HBA and TPA-HBA; Table S2. Bond Lengths for DMAC-HBA; Table S3. Bond Angles for DMAC-HBA; Table S4. Bond Lengths for TPA-HBA; Table S5. Bond Angles for TPA-HBA; Figure S5. Single Crystal and Cocrystal structure of DMAC-HBA and &Dioxane, TPA-HBA and & EtOH, with calculated Hydro-bond label below; Figure S6. Molecular orbital of Homo-Lumo and their Gap of DMAC-HBA &Dioxane, TPA-HBA & EtOH.; Table S6. Summary of electrochemical properties of DMAC-HBA and TPA-HBA; Figure S7. Cyclic Voltammograms for the (a) oxidation of DMAC-HBA and TPA-HBA and (b) oxidation behaviors of Ferrocene; Figure S8. transient PL decay spectra of DMAC-HBA and TPA-HBA measured in toluene solution (10^−5^ M) at 300 K & transient PL decay spectra of DMAC-HBA in different f_w_ THF/H_2_O.

## Sample Availability

Samples of the compounds are not available from the authors.
