# Supplementary material for: High-Contrast Visualization Chemiluminescence Based on AIE-Active and Base-Sensitive Emitters
Source: Molecules. 2023 May 8;28(9):3976. doi: 10.3390/molecules28093976 (PMC10180503; doi:10.3390/molecules28093976)
Supplement: Supplementary file 1 [file molecules-28-03976-s001.zip › molecules-2348055-supplementary.pdf]

Supporting Information

High-Contrast Visualization Chemiluminescence Based on AIE-Active and Base-Sensitive Emitters

Xiao-Wen Zhang [1,2,3,4], Xu-Lin Chen [1,2,3,*] and Can-Zhong Lu [1,2,3,4,*]

1 State Key Laboratory of Structural Chemistry, Fujian Institute of Research on the Structure of Matter, Chinese Academy of Sciences, Fuzhou 350002, China

2 Fujian Science & Technology Innovation Laboratory for Optoelectronic Information of China, Fuzhou 350108, China

3 Xiamen Key Laboratory of Rare Earth Photoelectric Functional Materials, Xiamen Institute of Rare Earth Mate-rials, Haixi Institutes, Chinese Academy of Sciences, Xiamen 361021, China

4 School of Physical Science and Technology, Shanghai Tech University, Shanghai 201210, China

* Correspondence: xlchem@fjirsm.ac.cn (X.-L.C.); czlu@fjirsm.ac.cn (C.-Z.L.)

Table of Contents

Figure S1. ^{1}H-NMR spectrum of DMAC-HBA (600 MHz, $CDCl_3$).

Figure S2. ^{13}C-NMR spectrum of DMAC-HBA (600 MHz, $CDCl_3$).

Figure S3. ^{1}H-NMR spectrum of TPA-HBA (600 MHz, $CDCl_3$).

Figure S4. ^{13}C-NMR spectrum of TPA-HBA (600 MHz, $CDCl_3$).

Table S1. Crystal data and structure refinements for DMAC-HBA and TPA-HBA.

Table S2. Bond Lengths for DMAC-HBA.

Table S3. Bond Angles for DMAC-HBA.

Table S4. Bond Lengths for TPA-HBA.

Table S5. Bond Angles for TPA-HBA.

Figure S5. Single Crystal and Cocrystal structure of DMAC-HBA and &Dioxane, TPA-

HBA and & EtOH, with calculated Hydro-bond label below.

Figure S6. Molecular orbital of Homo-Lumo and their Gap of DMAC-HBA &Dioxane, TPA-HBA & EtOH.

Table S6. Summary of electrochemical properties of DMAC-HBA and TPA-HBA.

Figure S7. Cyclic Voltammograms for the (a) oxidation of DMAC-HBA and TPA-HBA and (b) oxidation behaviours of Ferrocene.

Figure S8. transient PL decay spectra of DMAC-HBA and TPA-HBA measured in toluene solution (10^{-5} M) at 300 K & transient PL decay spectra of DMAC-HBA in different f_w THF/H_2O.

1. NMR Spectra

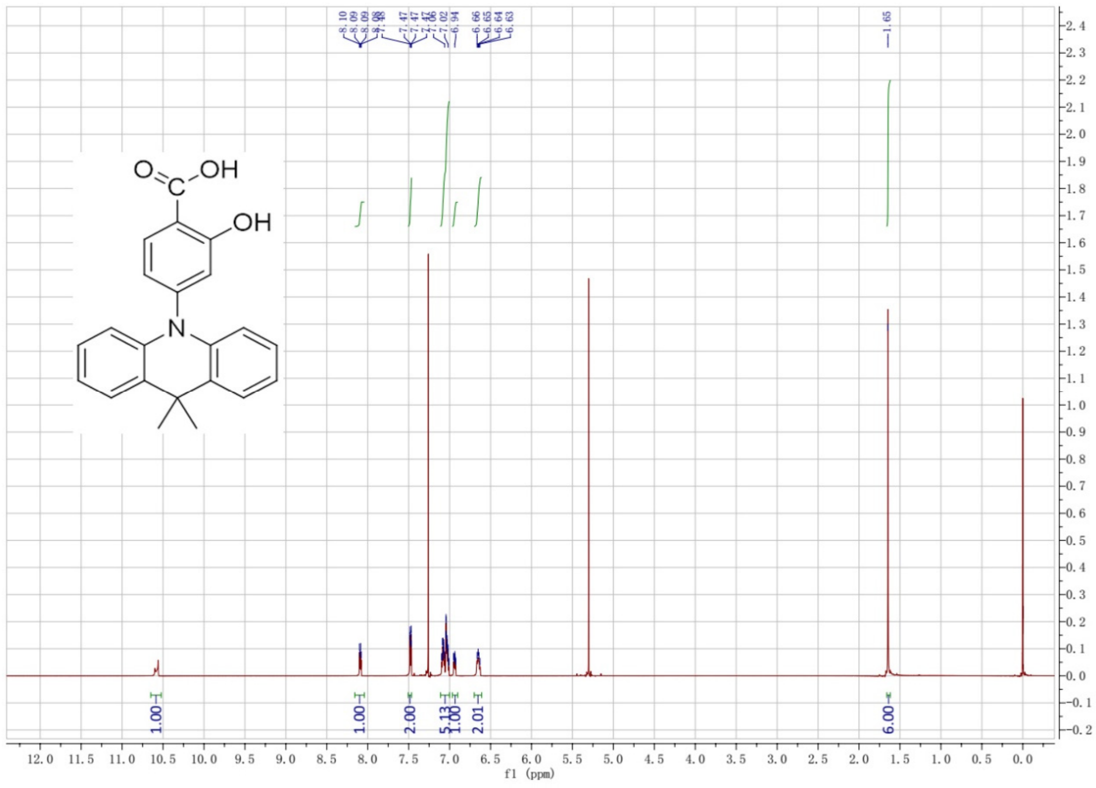

Figure S1. ^{1}H-NMR spectrum of DMAC-HBA (600 MHz, $CDCl_3$).

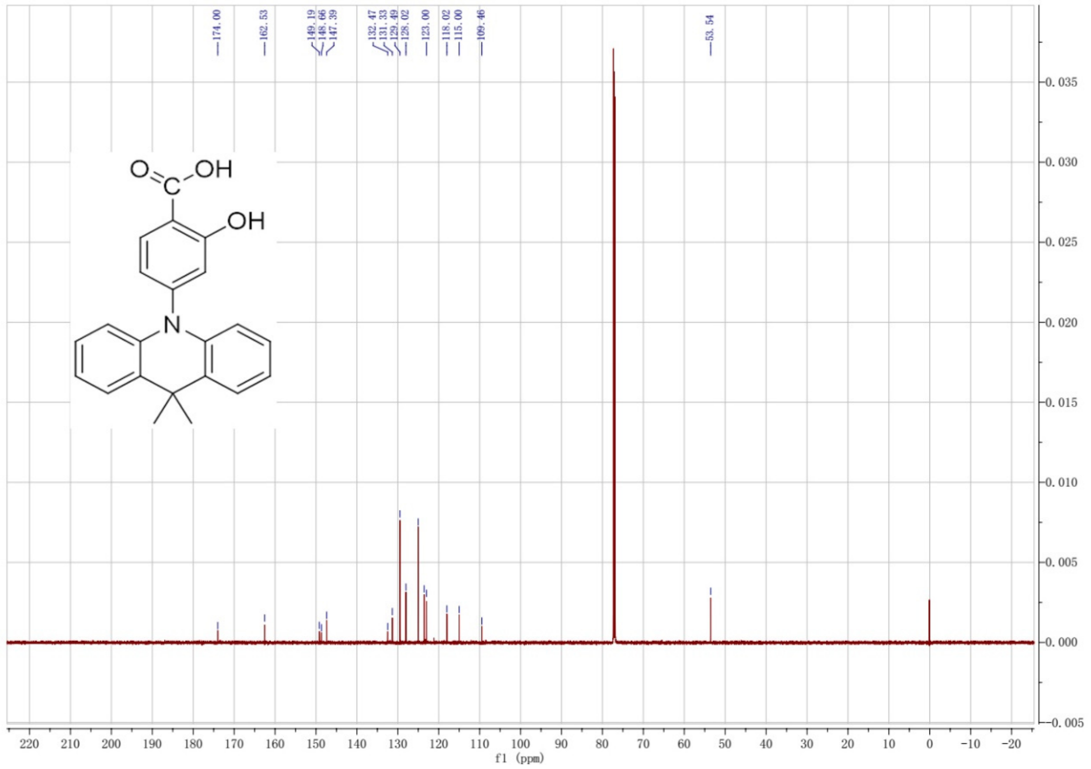

Figure S2. ^{13}C-NMR spectrum of DMAC-HBA (151 MHz, $CDCl_3$).

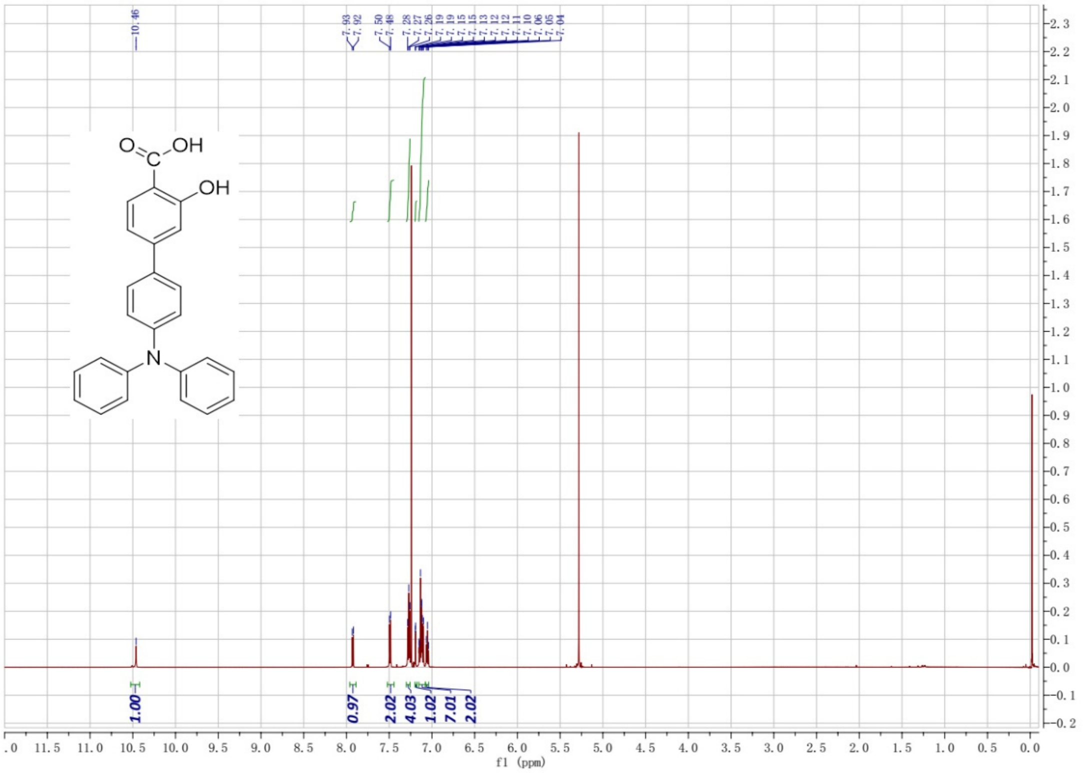

Figure S3. ^{1}H-NMR spectrum of TPA-HBA (600 MHz, $CDCl_3$).

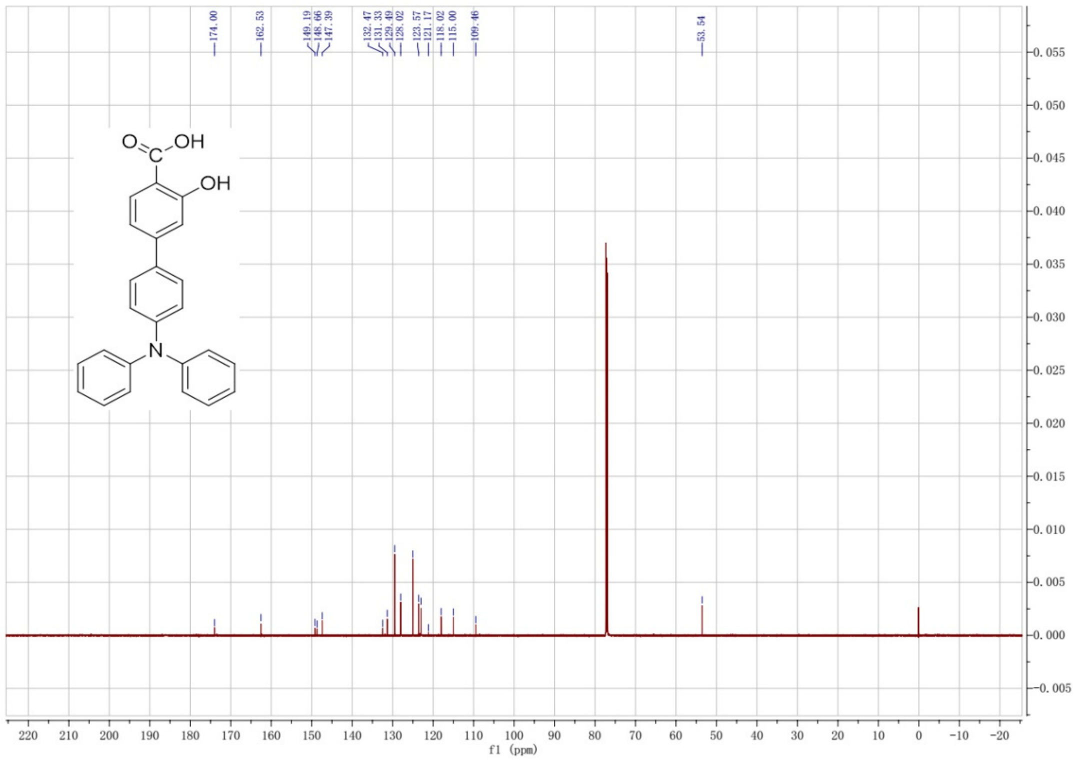

Figure S4. ^{13}C-NMR spectrum of TPA-HBA (151 MHz, $CDCl_3$).

2. X-ray Crystallographic Analysis

Table S1. Crystal data and structure refinements for DMAC-HBA and TPA-HBA.

Compounds	DMAC-HBA	TPA-HBA
Empirical formula	$C_{22}H_{19}NO_3$	$C_{25}H_{19}NO_3$
Formula weight	345.14	381.41
Temperature/K	200	200
Crystal system	monoclinic	tetragonal
Space group	P 1 21 1	P4(2)/n
a/Å	9.0126(3)	22.2520(11)

b/Å	30.5182(12)	22.2520(11)
c/Å	12.6586(5)	8.1795(5)
α/°	90	90
β/°	103.3060(10)	90
γ/°	90	90
Volume/Å^3	3388.26	4050.1(5)
Z	2	8
ρ_{calc}g/cm^3	0.8119	0.7533
μ/mm^{-1}	0.09	0.082
F(000)	1442.0	1600.0
Crystal size/mm^3	0.12 × 0.01 × 0.01	0.08 × 0.01 × 0.01
Radiation	MoKα (λ = 0.71073)	MoKα (λ = 0.71073)
2Θ range for data collection/°	2.322 to 31.076	2.589 to 27.476
Index ranges	-12≤ h ≤ 13, -44 ≤ k ≤ 44, -18 ≤ l ≤ 17	-27≤ h ≤ 28, -28 ≤ k ≤ 28, -10 ≤ l ≤ 10
Reflections collected	68893	30527
Independent reflections	21505[R_{int} = 0.1001, R_{sigma} = 0.1253]	4632 [R_{int} = 0.0754, R_{sigma} = 0.0459]
Data/restraints/parameters	21505/0/953	4632/0/307
Goodness-of-fit on F2	1.002	1.036
Final R indexes [I>=2σ (I)]	R_1 = 0.0706, wR_2 = 0.1453	R_1 = 0.0517, wR_2 = 0.1083
Final R indexes [all data]	R_1 = 0.1639, wR_2 = 0.1874	R_1 = 0.1035, wR_2 = 0.132
Largest diff. peak/hole / e Å-	0.33/-0.28	0.25/-0.20

Table S2. Bond Lengths for DMAC-HBA.

Atom	Atom	Length/Å	Atom	Atom	Length/Å
O001	C019	1.248(7)	C00R	C00V	1.404(8)
O002	C00J	1.242(7)	C00S	C01M	1.400(8)
O003	C00H	1.349(7)	C00S	C01W	1.528(8)
O004	C00J	1.325(7)	C00S	C024	1.417(8)
O005	C01V	1.352(6)	C00T	C01C	1.450(8)
O006	C00T	1.244(7)	C00U	C01E	1.406(8)
O007	C019	1.321(7)	C00U	C01T	1.416(8)
O008	C00Q	1.363(7)	C00V	C01G	1.416(8)
O009	C00Z	1.230(7)	C00W	C011	1.374(8)
O00A	C00N	1.358(7)	C00W	C01U	1.384(9)
O00B	C00Z	1.324(7)	C00X	C01K	1.386(8)
O00C	C00T	1.312(7)	C00Y	C01I	1.524(8)
N00D	C00I	1.431(7)	C00Y	C01O	1.540(8)
N00D	C00O	1.407(7)	C00Y	C028	1.522(8)
N00D	C00P	1.425(7)	C00Y	C02U	1.546(8)
N00E	C00V	1.397(7)	C00Z	C01B	1.468(8)
N00E	C00X	1.439(7)	C010	C02F	1.433(8)
N00E	C024	1.425(7)	C010	C02H	1.395(9)
N00F	C00U	1.392(8)	C011	C01K	1.381(8)
N00F	C015	1.411(8)	C013	C016	1.388(8)
N00F	C027	1.440(8)	C013	C023	1.529(8)
N00G	C010	1.367(8)	C013	C02E	1.380(9)
N00G	C014	1.437(8)	C014	C02I	1.385(8)
N00G	C016	1.419(8)	C014	C02Q	1.396(9)
C00H	C00L	1.404(7)	C015	C01J	1.396(8)
C00H	C00R	1.401(7)	C015	C01X	1.409(8)
C00I	C01H	1.394(8)	C016	C02G	1.407(8)

C00I	C01O	1.390(8)	C017	C027	1.360(8)
C00J	C00M	1.452(7)	C018	C01E	1.370(8)
C00K	C00X	1.395(8)	C018	C02L	1.397(9)
C00K	C01U	1.396(8)	C01A	C01X	1.387(9)
C00K	C01W	1.531(8)	C01A	C02N	1.372(9)
C00L	C019	1.456(7)	C01B	C01L	1.412(8)
C00L	C020	1.383(8)	C01C	C01V	1.416(8)
C00M	C00N	1.417(7)	C01C	C02B	1.387(8)
C00M	C01F	1.403(8)	C01D	C01Q	1.388(8)
C00N	C012	1.385(7)	C01F	C01P	1.364(8)
C00O	C012	1.404(8)	C01G	C020	1.376(8)
C00O	C01P	1.421(8)	C01H	C01Y	1.378(9)
C00P	C01D	1.386(8)	C01I	C02A	1.405(8)
C00P	C01I	1.396(7)	C01J	C026	1.509(8)
C00Q	C017	1.397(8)	C01J	C029	1.408(9)
C00Q	C01B	1.391(8)	C01L	C01S	1.382(9)
C01M	C01R	1.360(9)	C021	C02E	1.391(9)
C01N	C01W	1.547(9)	C021	C02K	1.370(9)
C01O	C02J	1.395(9)	C022	C02J	1.387(9)
C01Q	C02D	1.390(8)	C023	C02F	1.528(8)
C01R	C02C	1.392(9)	C023	C02O	1.536(9)
C01S	C027	1.394(9)	C023	C02V	1.535(9)
C01T	C01Z	1.388(9)	C024	C025	1.381(8)
C01T	C026	1.535(8)	C025	C02C	1.382(9)
C01V	C02I	1.393(8)	C026	C02T	1.542(9)
C01W	C02M	1.537(8)	C026	C02W	1.545(9)
C01Y	C022	1.363(9)	C029	C02N	1.371(9)
C01Z	C02L	1.388(10)	C02A	C02D	1.383(9)
C02B	C02Q	1.370(9)	C02H	C02P	1.386(9)

C02F	C02R	1.402(9)	C02P	C02S	1.399(10)
C02G	C02K	1.369(9)	C02R	C02S	1.370(10)

Table S3. Bond Angles for DMAC-HBA.

Atom	Atom	Atom	Angle/°	Atom	Atom	Atom	Angle/°
C5	N1	C6	122.4(5)	C34	C29	C30	112.0(5)
C5	N1	C14	122.6(4)	C34	C29	C33	109.3(5)
C14	N1	C6	113.4(4)	C32	C31	C24	122.4(5)
O6	C2	C24	123.3(5)	C31	C32	C26	119.5(6)
O6	C2	C25	115.4(5)	C35	C34	C29	124.1(5)
C25	C2	C24	121.3(5)	C35	C34	C39	118.4(5)
C26	N3	C27	123.2(5)	C39	C34	C29	117.3(5)
C26	N3	C39	122.2(5)	C36	C35	C34	120.0(6)
C39	N3	C27	112.7(4)	C35	C36	C37	121.4(6)
C49	N4	C48	117.7(5)	C38	C37	C36	119.8(6)
C60	N4	C48	119.0(5)	C37	C38	C39	118.9(6)
C60	N4	C49	121.6(5)	C34	C39	N3	117.8(5)
C71	N5	C70	118.6(5)	C38	C39	N3	120.6(5)
C78	N5	C70	119.8(5)	C38	C39	C34	121.4(5)
C78	N5	C71	121.6(5)	C41	C40	C28	119.9(6)
O1	C1	C88	115.4(5)	C42	C41	C40	120.5(6)
O2	C1	O1	121.4(5)	C41	C42	C43	120.6(6)
O2	C1	C88	123.2(5)	C27	C43	C42	118.8(5)
C3	C88	C1	120.7(5)	O7	C44	C45	115.8(5)
C22	C88	C1	122.6(5)	O8	C44	O7	121.4(6)
C22	C88	C3	116.6(5)	O8	C44	C45	122.8(6)
O3	C3	C88	122.0(5)	C46	C45	C44	120.7(6)

O3	C3	C4	116.2(5)	C46	C45	C53	118.8(5)
C4	C3	C88	121.7(5)	C53	C45	C44	120.5(5)
C3	C4	C5	120.0(5)	C47	C46	C45	120.8(6)
N1	C5	C21	120.3(5)	C46	C47	C48	120.0(6)
C4	C5	N1	121.0(5)	C47	C48	N4	118.2(5)
C4	C5	C21	118.7(5)	C54	C48	N4	121.2(6)
C7	C6	N1	116.4(5)	C54	C48	C47	120.5(6)
C20	C6	N1	120.5(5)	C50	C49	N4	120.3(5)
C20	C6	C7	122.8(5)	C50	C49	C65	120.2(6)
C6	C7	C10	118.4(5)	C65	C49	N4	119.4(5)
C8	C7	C6	117.1(6)	C49	C50	C51	120.0(5)
C8	C7	C10	124.4(5)	C62	C50	C49	118.0(5)
C9	C8	C7	121.9(5)	C62	C50	C51	121.4(5)
C19	C9	C8	119.6(5)	C50	C51	C	107.3(5)
C7	C10	C12	108.7(5)	C50	C51	C55	109.5(5)
C7	C10	C13	105.7(5)	C50	C51	C61	111.7(6)
C11	C10	C7	111.8(5)	C55	C51	C	107.6(6)
C11	C10	C12	107.5(5)	C61	C51	C	107.3(6)
C11	C10	C13	112.1(5)	C61	C51	C55	113.1(5)
C13	C10	C12	111.0(5)	O9	C53	C45	123.0(5)
C14	C13	C10	117.4(5)	O9	C53	C54	116.4(5)
C18	C13	C10	125.6(5)	C54	C53	C45	120.6(5)
C18	C13	C14	116.9(6)	C48	C54	C53	119.3(6)
C13	C14	N1	117.1(5)	C56	C55	C51	120.5(5)
C15	C14	N1	121.3(5)	C56	C55	C60	118.5(6)
C15	C14	C13	121.3(5)	C60	C55	C51	120.8(5)
C16	C15	C14	120.0(6)	C57	C56	C55	121.4(6)
C17	C16	C15	120.3(6)	C56	C57	C58	119.6(6)
C16	C17	C18	119.0(6)	C59	C58	C57	120.5(7)

C17	C18	C13	122.5(6)	C58	C59	C60	121.0(6)
C9	C19	C20	118.5(6)	N4	C60	C55	118.4(5)
C6	C20	C19	120.1(5)	N4	C60	C59	122.6(6)
C22	C21	C5	119.7(6)	C59	C60	C55	118.9(6)
C21	C22	C88	123.1(5)	C50	C62	C63	122.5(6)
O4	C23	C24	115.6(5)	C64	C63	C62	118.8(6)
O5	C23	O4	120.4(5)	C63	C64	C65	120.1(6)
O5	C23	C24	124.0(5)	C64	C65	C49	120.2(6)
C2	C24	C23	120.1(5)	O10	C66	C67	115.3(5)
C31	C24	C2	117.6(5)	O11	C66	O10	121.9(5)
C31	C24	C23	122.1(5)	O11	C66	C67	122.8(6)
C26	C25	C2	119.9(5)	C68	C67	C66	119.2(6)
N3	C26	C32	120.4(5)	C75	C67	C66	120.8(5)
C25	C26	N3	120.3(5)	C75	C67	C68	120.0(5)
C25	C26	C32	119.3(5)	C69	C68	C67	119.1(6)
C28	C27	N3	118.2(5)	C68	C69	C70	119.6(6)
C43	C27	N3	120.3(5)	C69	C70	N5	117.0(5)
C43	C27	C28	121.3(5)	C76	C70	N5	121.0(6)
C27	C28	C29	117.8(5)	C76	C70	C69	121.9(6)
C27	C28	C40	118.9(5)	C72	C71	N5	120.3(5)
C40	C28	C29	123.3(5)	C72	C71	C87	120.0(6)
C28	C29	C30	111.9(5)	C87	C71	N5	119.7(5)
C28	C29	C33	109.0(5)	C71	C72	C73	121.6(5)
C30	C29	C33	108.1(5)	C71	C72	C84	118.6(6)
C34	C29	C28	106.4(5)	C84	C72	C73	119.8(5)
C78	C77	C73	119.9(5)	C72	C73	C74	108.2(5)
C82	C77	C73	121.4(5)	C72	C73	C77	111.3(5)
C82	C77	C78	118.4(6)	C72	C73	C83	112.0(5)
N5	C78	C77	119.6(5)	C74	C73	C83	109.0(5)

N5	C78	C79	121.6(5)	C77	C73	C74	106.5(5)
C79	C78	C77	118.8(5)	C77	C73	C83	109.7(5)
C80	C79	C78	121.4(6)	O12	C75	C67	122.8(5)
C79	C80	C81	119.7(6)	O12	C75	C76	117.5(5)
C82	C81	C80	119.9(6)	C67	C75	C76	119.6(5)
C81	C82	C77	121.8(6)	C70	C76	C75	119.8(6)
C85	C84	C72	121.3(6)	C85	C86	C87	120.8(6)
C84	C85	C86	119.2(6)	C86	C87	C71	120.1(6)

Table S4. Bond Lengths for TPA-HBA.

Atom	Atom	Length/Å	Atom	Atom	Length/Å
O001	C007	1.320(4)	C00D	C2	1.39
O002	C007	1.236(4)	C00D	C1	1.39
O003	C00I	1.366(4)	C2	C4	1.39
N004	C005	1.418(4)	C4	C3	1.39
N004	C00D	1.542(18)	C3	C5	1.39
N004	C00F	1.420(4)	C5	C1	1.39
N004	C6	1.364(12)	C00E	C00G	1.392(5)
C005	C008	1.392(5)	C00F	C00L	1.394(5)
C005	C00H	1.385(5)	C00H	C00K	1.402(5)
C006	C007	1.459(5)	C00I	C00J	1.386(5)
C006	C00E	1.410(5)	C00K	C00O	1.386(6)
C006	C00I	1.397(5)	C00L	C00M	1.406(5)
C008	C00N	1.394(5)	C00N	C00O	1.398(6)
C009	C00A	1.477(5)	C6	C1A	1.420(12)
C009	C00C	1.387(5)	C6	C2A	1.437(12)
C009	C00M	1.406(5)	C1A	C5A	1.402(12)
C00A	C00G	1.422(5)	C5A	C3A	1.432(16)

C00A	C00J	1.395(5)	C3A	C4A	1.416(17)
C00B	C00C	1.407(5)	C4A	C2A	1.426(12)
C00B	C00F	1.404(5)	.		

Table S5. Bond Angles for TPA-HBA.

Atom	Atom	Atom	Angle/°	Atom	Atom	Atom	Angle/°
C005	N004	C00D	114.3(9)	C2	C00D	N004	121.1(11)
C005	N004	C00F	119.4(3)	C2	C00D	C1	120
C00F	N004	C00D	124.9(9)	C1	C00D	N004	118.8(11)
C6	N004	C005	124.5(9)	C4	C2	C00D	120
C6	N004	C00F	115.8(9)	C2	C4	C3	120
C008	C005	N004	120.1(3)	C4	C3	C5	120
C00H	C005	N004	119.1(3)	C3	C5	C1	120
C00H	C005	C008	120.8(3)	C5	C1	C00D	120
C00E	C006	C007	120.5(3)	C00G	C00E	C006	121.0(3)
C00E	C006	C00I	118.7(3)	C00B	C00F	N004	119.9(3)
C00I	C006	C007	120.7(3)	C00L	C00F	N004	119.6(3)
O001	C007	C006	115.8(4)	C00L	C00F	C00B	120.6(3)
O002	C007	O001	121.4(3)	C00E	C00G	C00A	119.1(4)
O002	C007	C006	122.8(3)	C00K	C00H	C005	119.3(4)
C00N	C008	C005	119.3(3)	O003	C00I	C006	122.2(3)
C00C	C009	C00A	120.5(3)	O003	C00I	C00J	116.7(3)
C00C	C009	C00M	119.2(3)	C00J	C00I	C006	121.2(4)
C00M	C009	C00A	120.3(3)	C00A	C00J	C00I	120.1(3)
C00G	C00A	C009	119.3(4)	C00O	C00K	C00H	120.6(4)
C00J	C00A	C009	120.8(3)	C00M	C00L	C00F	119.4(3)
C00J	C00A	C00G	119.9(3)	C00L	C00M	C009	120.6(4)
C00C	C00B	C00F	119.1(4)	C00O	C00N	C008	120.6(4)
C009	C00C	C00B	121.0(3)	C00K	C00O	C00N	119.3(4)

N004	C6	C1A	118.4(12)	C1A	C5A	C3A	121.8(11)
N004	C6	C2A	117.2(11)	C5A	C3A	C4A	120.0(9)
C2A	C6	C1A	124.3(10)	C3A	C4A	C2A	120.7(10)
C5A	C1A	C6	116.6(10)	C6	C2A	C4A	116.6(11)

3. Cocrystal structure, hydro-bond energy and FTO analysis

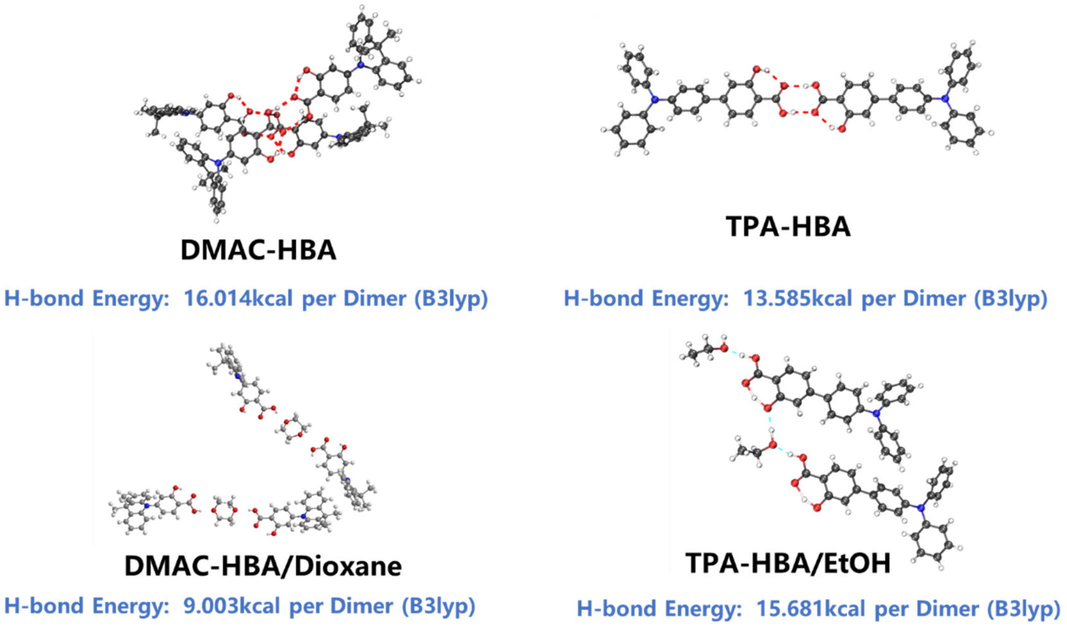

Figure S5. Single Crystal and Cocrystal structure of DMAC-HBA and &Dioxane, TPA-HBA and & EtOH， with calculated Hydro-bond label below.

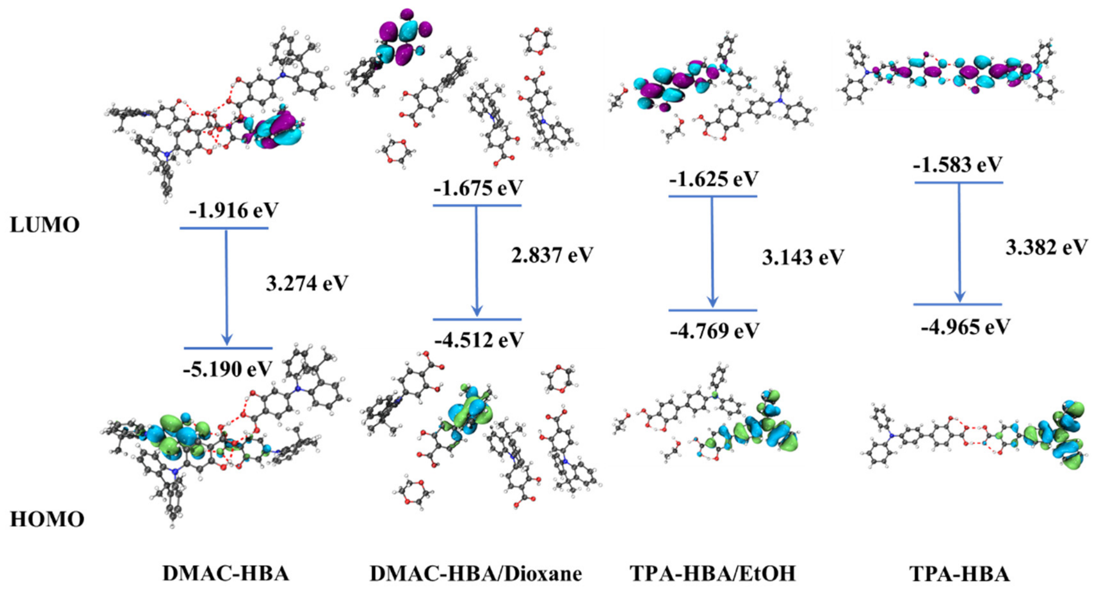

Figure S6. Molecular orbital of Homo-Lumo and their Gap of DMAC-HBA and &Dioxane, TPA-HBA & EtOH.

4. Electrochemical Properties

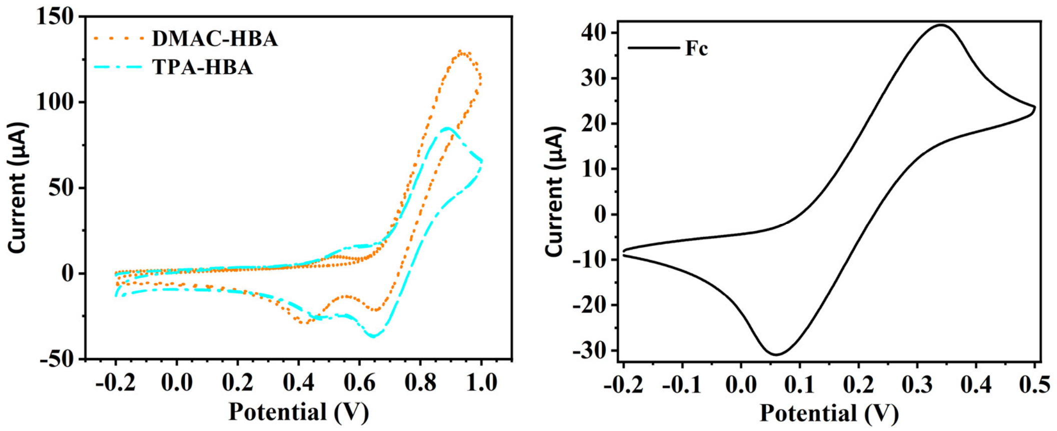

Figure S7. Cyclic Voltammograms for the (a) oxidation of DMAC-HBA and TPA-HBA and (b) oxidation behaviours of Ferrocene.

The CV measurements were carried out in anhydrous and nitrogen-saturated dichloromethane (DCM) solutions with 0.1 M n-Bu_4NPF_6 and 1.0 mM investigated compounds. Using glassy carbon electrode as working electrode, platinum wire as auxiliary electrode, porous glass wick $Ag/AgNO_3$

as reference electrode and ferrocene/ferrocenium as the internal standard. The HOMO energy level was calculated from the onset potential of oxidation by cyclic voltammetry.

$$[HOMO = - (4.8- E_{1/2(Fc/Fc+)} + E_{onset})]$$

The LUMO energy level was determined from the difference between the HOMO levels and optical band gap (E_g) estimated from the onset of the UV-Vis absorption band.

$E_g = 1241 / \lambda_{onset}$ $\quad\quad$ $[LUMO = HOMO + E_g]$

Table S6. Summary of electrochemical properties of DMAC-HBA and TPA-HBA.

**Compound**	**HOMO [a] (eV)**	**LUMO [b] (eV)**	**E_g [c] (eV)**
**DMAC-HBA**	**-5.02**	**-2.26**	**2.76**
**TPA-HBA**	**-5.02**	**-2.17**	**2.85**

(a) Obtained from the CV curves. (b) Calculated from the E_g and HOMO levels. (c) Optical energy gaps (E_g) were determined from the UV-Vis absorption spectra.

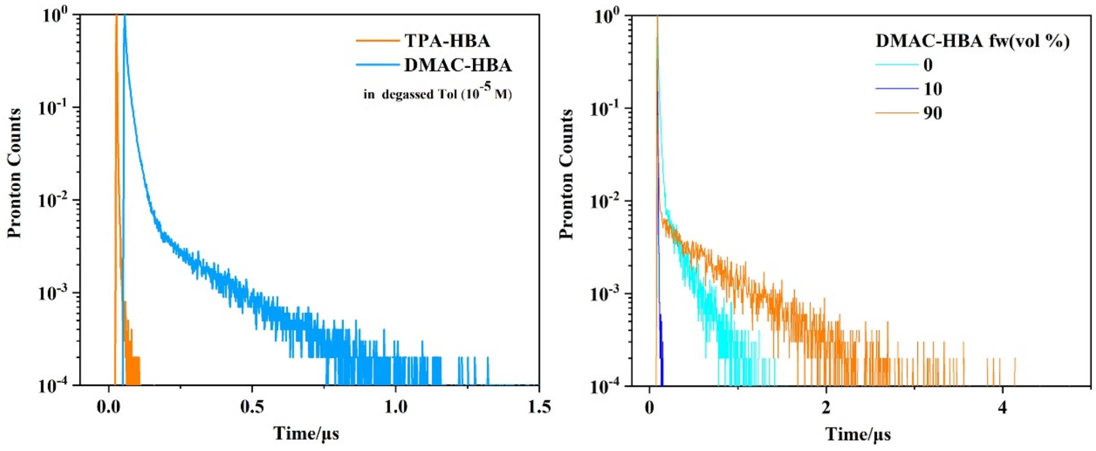

Figure S8. transient PL decay spectra of DMAC-HBA and TPA-HBA measured in toluene solution (10^{-5} M) at 300 K (left) & transient PL decay spectra of DMAC-HBA in different f_w THF/H_2O (right).
